# A qualitative study: Barriers and support for participation for children with disabilities

**DOI:** 10.4102/ajod.v3i1.112

**Published:** 2014-11-25

**Authors:** Anne Marie Witchger Hansen, Musonde Siame, Judith van der Veen

**Affiliations:** 1 Department of Occupational Therapy, Duquesne University, United States of America; 2 Cheshire Homes Society of Zambia, CBR Programme, Zambia; 3 Inclusive Development, CBM International, South Africa

## Abstract

**Background:**

This qualitative–exploratory study examined the barriers to participation amongst children with disabilities in Lusaka, Zambia, from the mothers’ perspective.

**Objectives:**

The objectives of this study were to understand how mothers of children with physical and cognitive disabilities who engaged their children in community-based rehabilitation (CBR) services in Lusaka, Zambia, perceived and described (1) the level of support they received and the barriers they encountered in terms of their child’s meaningful social participation; (2) the use and awareness of these barriers to identify and pursue advocacy strategies; and (3) hopes for their child’s future.

**Methods:**

Data were collected through semi-structured interviews with each mother in her home. Results: Findings revealed both support and barriers to the child’s social participation in relationship to their family, friends and community. Support also came from the CBR programme and mothers’ personal resourcefulness. Mothers identified their child’s school, their immediate environment and financial burdens as barriers to participation as well as their own personal insecurities and fears. Strategies to overcome barriers included internal and external actions. The mothers involved in the study hope their child’s abilities will improve with continued CBR services. Some mothers described a bleak future for their child due to a lack of acceptance and access to education.

**Conclusion:**

The findings of this study suggest the significant role the mother of a child with a disability plays in her child’s social participation. Recommendations include enhancing CBR programming for families, especially for mothers, and advocating on behalf of children with disabilities and their families to attract the attention of policy makers.

## Introduction

The United Nations Children’s Fund (UNICEF 2013) estimates that between 5% and 10% of all children in Africa are children with disabilities; children with disabilities are particularly vulnerable and influenced by the extent of their impairment as well as by the sex of the child. Research focussing on children with disabilities in developing countries suggests that 90% of these children do not attend school and are thus less likely to engage in other opportunities for social participation (Global Partnership for Children 2012).

This study was conducted in Lusaka, the capital and the largest city of Zambia. It is one of the fastest developing cities in southern Africa (World Association of Business Administration and Management Professionals 2013); Lusaka is the centre of commerce and government in Zambia. The population of Zambia is 13 million people (Central Statistics Office 2011), predominantly composed of indigenous African people speaking a variety of Bantu languages as well as English, which is the principal medium of communication. The majority persons with disabilities in Zambia live in poverty and generally have low literacy levels disproportionately compared to persons without disabilities (Sakala & Korpinen 2013). Educating children with disabilities remains a challenge for Zambia. An understanding of the practice of inclusive education is limited, and although the education of children with disabilities is guaranteed through a number of government policies and legislation (most notably the *Education Policy* and the *Zambia Disabilities Act of 1996*), recent studies have shown very high drop-out and low progression rates for children with disabilities (Sightsavers 2013). The Ministry of Education has indicated that children with disabilities constitute 5.1% of all learners in grades 1–9, but just 1.58% of enrolment for grades 10–12 (Sightsavers 2013). The Sixth National Development Plan of 2011 (SNDP) has recognised the need to enhance the inclusion of Learners with Special Education Needs (LSEN) in the mainstream school system (Sightsavers 2013).

The objectives of this study were to understand how mothers of children with physical and cognitive disabilities who engaged their children in community-based rehabilitation (CBR) services in Lusaka, Zambia, perceived and described:

- The level of support they received and the barriers they encountered in terms of their child’s meaningful social participation.
- The use and awareness of these barriers to identify and pursue advocacy strategies.
- Expectations for their child’s future.

The CBR programme serves a population of approximately 350 000 individuals. Staff members work with children and adults with all types of disabilities. They provide supervision and support to community-based rehabilitation workers (CRWs). A CRW seeks out people with disabilities who can benefit from ability restoring surgeries, therapeutic interventions and educational services as well as home-based rehabilitation services (Blind Christian Mission n.d.).

### Theoretical framework

The World Health Organization (WHO 2014) defines disability as ‘impairments, activity limitations and participation restrictions’ including ‘interactions between individuals with a health conditions and personal and environmental factors’. The theoretical framework for this study is based on the International Classification of Functioning (ICF) which shifts the focus away from the cause of a disability to the impact the disability has on the lives of people in society (WHO 2014). The ICF addresses the social model of disability, which regards disability as a social problem and not an individual’s attribute or impairment (Forsyth *et al*. 2007).

In the ICF study, participation is considered a chief indicator of child health, with or without regard for functional ability or diagnosis (Law *et al*. 2006). Children with disabilities are vulnerable to limited participation, which results from the interaction between children and their physical and social environments (Forsyth & Jarvis 2002; Law *et al*. 2006). The significance of this study lies in the examination of the level of support received and the barriers encountered in terms of the social participation amongst children with physical disabilities in Lusaka, Zambia, according to their mother’s perspective.

The social action goal is to define barriers to full participation for mothers of children with disabilities for future action with the participants of this study. The action component includes efforts to realise fuller participation in a manner that is consistent with the lived experiences of barriers and in ways that ensure that the basic rights of children with disabilities are met and that they are treated with human dignity.

### Literature review

Although a few studies explore mothers’ experiences of caring for children with disabilities in the African context (Gona *et al*. 2011; McNally & Mannan 2013), no studies were found that address mothers’ perspectives of the barriers they encountered and the level of support they received in terms of their children’s social participation. Several researchers explored environmental factors that impact social participation (King *et al*. 2003; Law *et al*. 2007). Bedell *et al*. (2013) examined patterns of community participation and environmental factors that affect community participation for school-aged children with and without disabilities in the United States of America and Canada, and found that stronger efforts are needed to support community participation of school-aged children.

#### Participation patterns amongst children with disabilities

Researchers discovered that children with disabilities tend to be more restricted in their participation and in the scope of their daily activities (e.g. formal and informal leisure and recreational activities outside of school, household tasks and social engagements) than their peers (Bedell *et al*. 2013; Brown & Gordon 1987; Hanvey & Avard 1994; McDougall *et al*. 2004). Children with disabilities often feel socially isolated (Anderson, Clarke & Spain 1982; Blum *et al*. 1991; Cadman *et al*. 1987; La Greca 1990; Law & Dunn 1993).

CanChild Centre for Childhood Disability Research studied 427 children between the ages of 6 and 14 years with physical disabilities to examine their patterns of participation and to determine the child-specific, family and environmental factors that influenced their participation in formal and informal activities (Law *et al*. 2006; King *et al*. 2009). They found that some factors are direct predictors of participation and some are more indirect predictors of participation (Law *et al*. 2006). Child and family preferences were found to be important predictors of children’s participation following adjustment for the child’s functional ability. They found that factors such as family cohesion and parental perceptions of environments were relatively unsupportive, and supportive relationships for the child indirectly influenced child participation (Law *et al*. 2006).

#### Direct and indirect predictors of participation

Direct predictors of participation include the child’s functional ability (i.e. cognitive, communicative and physical functioning), family participation in social and recreational activities, family values related to intellectual and cultural activities and child preferences for activities (Law *et al*. 2006). Indirect predictors of participation include parents’ perceptions of environmental barriers, family cohesion and supportive relationships for the child and family income. They also found that supportive (i.e. accessible, accommodating, socially supportive and nondiscriminatory) and resource-ready environments influenced participation through their effects on children’s functional ability. Greater social support from friends, parents, and teachers also enhanced participation by affecting children’s activity preferences. They concluded that families play an important role in providing opportunities, support and encouragement for children to take part in various activities (Law *et al*. 2006).

#### Participation in school and community

Participation in school activities is one of the most important outcomes of children’s inclusion in mainstream schools (Eriksson 2005). However, researchers found that children with disabilities in North America still face restricted participation in comparison with their nondisabled peers (Pitt & Curtin 2004; Richardson 2002).

Harding *et al*. (2009) interviewed six children with disabilities in Canada to gain a better understanding of how they view their participation in out-of-school-time activities in a range of environmental settings. The children identified aspects of their environments and activities that acted as supports or barriers to their participation. Environment accessibility and physical comfort were noted as supports. Social support came in the form of friends, friendly helpers, pets and neighbours. Lastly, participants’ health statuses were considered a barrier to their participation in some settings (Harding *et al*. 2009).

#### Community participation

Bedell *et al*. (2013) examined patterns of community participation and environmental factors that affect community participation for school-aged children with and without disabilities in the United States and Canada, and found that stronger efforts are needed to support community participation of these children.

### Conclusion

It is vital that parents, service providers and policy makers concerned with children with disabilities understand the barriers to and supports for participation (King *et al*. 2003; Law *et al*. 2006). This study will explore this issue of social participation for children with disabilities in Zambia.

## Research method and design

This is a qualitative–exploratory study that allowed researchers to explore the support for and barriers to children with disabilities’ social participation from their mother’s perspective. The qualitative methods are phenomenological in nature (Benner 1994) as this study explored the lived experience of mothers. The design used participatory action research methodologies (Minkler & Wallerstein 2003; Patton 2002) as it was a collaborative process that equitably involved all partners including mothers, CBR staff and researchers in the research process, and recognises the unique strengths that each stakeholder brings. A further purpose of this qualitative, community-based participatory research was to gain knowledge about social participation of children with disabilities to move into action for social change to improve community programmes to eliminate the difficulties they face.

The specific research questions that guide this research study are as follows:

- How do mothers of children with physical and cognitive disabilities perceive and describe the level of support they receive and barriers to their child’s meaningful social participation?
- How do mothers of children with physical disabilities perceive and describe their understanding of these barriers to identify and pursue advocacy strategies to address these barriers?
- How do mothers of children with disabilities perceive and describe their expectations for their child’s future?

### Participants and setting

Eleven mothers of children with physical disabilities were interviewed. In two cases, the mother had passed away and a sister of the mother became the primary caregiver who in both cases was interviewed. Mothers’ ages ranged from 24–45. Their children with disabilities included a total of ten boys and one girl, whose ages ranged from 2–21 and had a diagnosis which included cerebral palsy, spina bifida, cerebral malaria and spinal muscle atrophy. All children had participated in CBR services at some point (see Table 1a, Table 1b and Table 1c). CBR offers intervention programmes to support inclusive education, livelihoods, health and rehabilitation and support for activities of daily living (ADL). Mothers of children with disabilities who participated in this study described the daily occupations of their children in terms of (see Table 1c):

- communication
- activities of daily living
- mobility
- play
- school or education
- community activities.

**TABLE 1a:** Demographics of the children with disabilities.

Age	Number	Sex	Number	Impairment	Number	Cognition	Number
2-5	2	Male	10	Celebral	7	Intact	5
				Palsy			
6-10	4	Female	1	Spina Bifida	2	Impaired	5
11-15	3	-	-	Cerebral	1	-	-
				Malaria			
19-21	2	-	-	Spinal	1	-	-
				Muscle			
				Atrophy			

**TABLE 1b:** Demographics of the children with disabilities.

CBR Involvement	Age	Number	Living situation	Number
Began CBR Services	Before 8 months	5	Lives with immediate family	10
	After 8 months	2	Lives with extended family	1
	Not given	4	-	-
Duration of services	Greater than 5 years	2	-	-
	Less than 5 years	2	-	-
	Not given	7	-	-

CBR, community-based rehabilitation

**TABLE 1c:** Demographics of the children with disabilities.

Activity	Daily occupations	Number
Communication	Able to talk	6
	Speaks few words	1
	Unable to talk	3
	NA (2 year-old)	1
Mobility	Able to walk	3
	Able to walk with support	2
	Not able to walk	6
ADLs	Independence	6
	Partial independence	2
	Dependent	3
Play	Plays with self	6
	Plays with others	5
School	Attends school	5
	Does not attend school	6
Community activities	Participates in activities	6
	Does not participate in activities	5

ADLs, activities of daily living.

#### Data collection

Interviews were between 30 and 60 minutes and took place in each respondent’s home in various villages in the Lusaka area. Interviews were conducted in the local language and in English by the research team comprising the primary researcher, coresearcher and a community health worker. Researchers followed a semistructured interview guide.

#### Data analysis

Data analysis began by using a process of open coding. Initially data was organised qualitatively into open (broad) codes by analysing the responses to the interview questions. Continued comparison of data within and across interviews allowed the researcher to reduce the data into categories. Central ideas were refined as concepts, and the properties and dimensions of these concepts were identified in such a way that they were delineated; the range of properties of any given category were specified and grouped together (Patton 2002).

## Results

Findings of this study revealed that mothers of children with disabilities perceived and described family, friends and community as both support for and barriers to the child’s social participation. They also described their personal resourcefulness, staff members and services at the CBR as support. Mothers identified schools, the environmental context and financial burdens as barriers to participation, on one hand, and their own personal weaknesses, insecurities and fears, on the other. In addition, mothers described strategies to overcome barriers in terms of their internal and external actions. Thus, many mothers described internal strategies such as prayer, a deep faith and trust in God and maintaining a positive attitude as a form of internal action. External strategies, on the other hand, included joining a support group, learning more about disability to better understand their child’s condition, encouraging their child to be more independent in dressing, feeding and playing with other children, and seeking more CBR services.

When asked to describe their expectation for their child’s future, mothers hoped that their child’s abilities would improve and that they would receive support from the CBR programme in the form of equipment and more services. However, others described the future of their children as bleak due to a lack of acceptance and access to education.

### Support to social participation

Mothers claim that their support for their child’s social participation is the people and services that encourage the child to engage in social activities in various contexts.

### Family

Some mothers described their family as supportive in terms of their child’s social participation; for example, some family members accept the child with a disability and welcome the child to participate in family celebrations and other activities. One mother stated the following: ‘Most family members accept the condition of my son. They invite us to family celebrations. That gives me strength to commit myself to his condition and to improve’ (Mother of PP, 11-year-old child with cerebral palsy).

### Friends and/or peers

A few mothers described their child’s friends as accepting of the child’s condition and, as a result, they play together. Several mothers reported that younger children in the neighbourhood came to their house to play: ‘When playing with friends he can do more things – like he can do a somersault when crawling and sort out objects and play with toy cars’ (Mother of KB, 6-year-old child with brain damage and cerebral palsy).

### Community and/or neighbours

Several mothers described the community, particularly their church, as being supportive and welcome their children to participate in Sunday services; for instance, one mother told a story of how community members used to laugh at her child’s condition during his first few years of his life, but has now come to accept him: ‘Neighbours used to treat him as abnormal but with explanation they understand and accept him as “one of the kids”’ (Mother of MS, 2-year-old child with cerebral palsy).

### Community-based rehabilitation staff and services

Mothers described the CBR as a strong support for their child’s social participation. Most mothers expressed appreciation for CBR services that support their child’s participation such as movement and mobility therapy, orthopaedic equipment, as well as educational and emotional support:

‘After CBR he can walk better and his arm is in a better position than before. He can then interact with friends and play with them. His attitude has changed too Now he can walk and move around, making life easier. There has been a big change, walking was a problem before, but now he can walk better, and his arm is in a better position than before. He can interact with his friends and play with them. He plays daily without any problem.’ (Mother of MS, 2-year-old child with cerebral palsy)

Many mothers described CBR support groups as offering encouragement to their child and provide hope for the future.

### Personal resourcefulness

Several mothers described their own resourcefulness as strength. Apart from belonging to support groups, one mother also described her livelihood activities as a support to her child’s participation:

‘By engaging in income generating activities, I feel as if I can help my child engage in more activities and help myself by paying school fees and paying for food.’ (Mother of MP, 11-year-old child with spina bifida)

### Barriers to social participation

The research established that barriers to social participation for children with disabilities are in the form of people and services that prevent or discourage these children from engaging in activities.

#### Family

Some mothers claimed family members are a barrier to their child’s social participation as they do not accept the child: ‘Our families live close by. However, they do not invite us to celebrations. They family never accepted our son. I think they fear him!’ (Mother of MP, 11-year-old child with spina bifida).

Several mothers reflected how their extended family members or the fathers of the children do not accept their disability and therefore would not help with the child’s care: ‘We have no family members near they live far away and only come to visit us when major problems happen’ (Mother of TM, 8-year-old child with cerebral palsy).

In addition: ‘His father does not accept him! He will not help me take care of him.’ (Mother of FH, 4-year-old child with spinal muscle atrophy)

In some situations, one side of extended family members blames the other family members for the disability:

‘My family and my husband’s family blame another family for my son’s disability. When they are together, they argue about whose fault it is. No one in either family seems to understand disability.’ (Mother of MI, 19-year-old child with cerebral palsy)

#### Friends

A few mothers described their children’s friends as a barrier. One mother told a story of how peers beat her child whilst her other children were carrying him to a CBR institution: ‘One day when his siblings were taking him to the rehab center, they ran into mean kids. His siblings escaped but left him behind. His friends beat him up!’ (Mother of MI, 19-year-old child with cerebral palsy).

#### Community and/or neighbours

Many mothers claimed their communities do not accept their child and, as a result, did not take them to community events:

‘*People in the community focus on his disability and laugh at him, so in those early days*, I stopped taking him to any community activities except to church. Some do not take their child to church in fear of being shunned.’ (Mother of MI, 19-year-old child with cerebral palsy)

#### Schools

Many mothers described the school environment and teachers as major barriers to their children’s lack of social participation; for example, a mother described how her disabled son’s teacher told her that he does not have any potential for the future and thus should not take up classroom space:

‘I took him to school but they told me they cannot give him space because they are not equipped to handle a child with a disability. As I was leaving the school she called out that children like mine do not belong in school!’ (Mother of LM, 10-year-old child with cerebral palsy)

Several mothers described how head masters of schools accepted their children into their schools, yet the teachers refused to allow children with disabilities into the classroom, claiming they did not have the equipment or skills to accommodate them.

#### Environment

Many mothers described the rough condition of the roads in their villages, intense traffic patterns in the city and the inaccessible public transport systems as barriers to their children’s social participation. One mother described how hard it is to transport her child in his wheelchair to church, only to go back after wheeling him a short distance as a result of the numerous potholes in the road’s surface and the increasing weight of her 11-year-old son: ‘It is too far to push him in his wheelchair to school, church or social events on this very rough dirt road. We rarely go anyplace, we stay home’ (Mother of FH, 4-year-old child with spinal muscle atrophy).

#### Financial burdens

Many mothers told stories of financial burden that resulted from the additional expenses of raising a child with a disability. They often lamented that they have little time to earn money whilst caring for their child which requires them to be home all day:

‘Fees for health services from my child, school fees and transportation fees to and from the hospital are a huge financial burden. I take care of my son all day long, and do not have time to find paid work.’ (Mother of TM, 8-year-old child with cerebral palsy)

#### Lack of awareness and understanding of disability related issues

Several mothers claimed their families lack awareness regarding disability and therefore do not understand issues related to disability and are afraid of being near a child with disabilities: ‘Some people in our community ask me questions, like“why are you carrying your son” and “why doesn’t he talk?”. They don’t seem to understand disability’ (Mother of KB, 10-year-old child with cerebral palsy).

#### Personal weaknesses

Many mothers described themselves as a barrier to their own children’s social participation; for example, some mothers indicated that they feel alone and isolated. They expressed both emotional and physical burden: ‘My problems seem so big and the burden is great and falls on me. Everything falls on me!’ (Mother of MS, 10-year-old child with cerebral palsy).

In addition:

‘He needs so much more attention than the other children. I do not have a strong support system, so I feel I need to be near him and with him at all times.’ (Mother of PP, 11-year-old child with cerebral palsy)

Many of these mothers described how uncomfortable they feel when trying to explain to family and friends what it takes to raise a child with a disability. Some mothers feel they lack the basic skills needed to overcome these barriers:

‘I feel stuck! I do not know what to do to advocate for my son. I do not know how to read or write. It is difficult to communicate with others about the burden of his physical condition.’ (Mother of MS, 10-year-old child with cerebral palsy)

Several mothers described their reluctance to take their children to community activities because they fear that their children can fall and hurt themselves, and that they can be disruptive to their peers: ‘I cannot take him to church or other places in the community because I fear he will be disruptive and he is too heavy to carry’ (Mother of PP, 11-year-old child with cerebral palsy).

Mothers of children aged between 10 and 15 years also complained of their children being too heavy to carry during activities.

#### Strategies to overcome barriers

Mothers of children with disabilities described their strategies to overcome the barriers to their child’s social participation as internal actions and external actions. Internal strategies are personal, intrinsic actions which mothers employed to address the barriers whilst attempting to engage their children in home, school or community activities. Many mothers described prayer and a deep faith and trust in God as an internal strategy that gives them strength to handle the challenges they face: ‘My strength and inspiration comes from God. Without prayer and my faith, I could not do this!’ (Mother of PP, 11-year-old child with cerebral palsy).

In addition: ‘I stay strong by the grace of God!’ (Mother of TM, 8-year-old child with cerebral palsy).

Many mothers discussed how they keep a positive attitude and rely on their internal strength to handle the struggles they face as well as strength from other parents: ‘To take care of him, I had to learn to accept him and accept that he had problems. This helped make things easier’ (Mother of MS, 2-year-old child with cerebral palsy).

In addition: ‘I spend time with other parents to talk and think positively.’ (Mother of FH, 4-year-old child with spinal muscle atrophy)

External strategies are outward actions, which mothers described they employ in an attempt to address the barriers they experience when they engage their children in home, school or community activities. Mothers described their outward actions as seeking support from other parents of children with disabilities by organising or joining a support group: ‘I have been reaching out for spiritual support from my church. I joined a support group there that really helps me feel that I am not alone’ (Mother of MI, 19-year-old child with cerebral palsy).

In addition: ‘I formed my own support group with other moms of kids with disabilities; now I know that I am not alone!’ (Mother of MS, 2-year-old child with cerebral palsy).

Mothers described personal actions such as educating themselves on disability related issues to improve their understanding of their children’s condition: ‘I have been learning about health and nutrition for my son, and what to expect with his conditions and his disability’ (Mother of MS, 2-year-old child with cerebral palsy).

Several mothers described how they encourage their children to be more independent such as children being able to dress and feed themselves, and by playing with other children: ‘I tell my son he has to take care of his things, such as putting things away and become more independent!’ (Mother of MA, 2-year-old child with cerebral palsy).

In addition: ‘I tell him to “go play” with your friends! Don’t stay at home all day!’ (Mother of MA, 10-year-old child with cerebral palsy).

Many mothers address the barriers to participation their children face by seeking more CBR services:

‘I will continue to take my son to rehab to help him with his mobility. I hope they can help him learn to do more and take care of himself, too.’ (Mother of MA, 10-year-old child with cerebral palsy)

#### Expectations for the future

Many of the mothers emphasised their expectations in terms of their children’s future. Most mothers perceived and described their child’s future in terms of their expectation that their child’s abilities will improve: ‘I hope my child will stand and walk in the future’ (Mother of FH, 4-year-old child with spinal muscle atrophy).

In addition: ‘I hope he will become more independent in the future so he can take care of me in my old age’ (Mother of MI, 19-year-old child with cerebral palsy).

When describing these expectations for the future, most of these mothers included their expectations for more intensive CBR services such as teaching their children to walk, to become independent and to provide more therapeutic exercises: ‘He needs more intensive exercises at CBR so that he can walk. If he could walk we could find him a school’ (Mother of KB, 6-year-old child with brain damage and cerebral palsy).

In addition: ‘We need CBR to teach him to become more independent in his ADLs’ (Mother of TM, 8-year-old child with cerebral palsy).

Their hopes also included more equipment for their child:‘I really need a wheelchair to take him out to school and church’ (Mother of FH, 4-year-old child with spinal muscle atrophy).

In addition: ‘I would like a conventional wheelchair with a tray for him to write on’ (Mother of DB, 15-year-old child with spina bifida).

Most mothers hope that CBR staff will assist them with finding and funding their child’s education including tertiary education and eventually to find employment for their child:‘I would like to have CBR place him in a school that meets his needs’ (Mother of MA, 10-year-old child with cerebral palsy).

In addition: ‘I need CBR to help pay for his schooling’ (Mother of MP, 11-year-old child with spina bifida); and ‘I want him to go to college and for CBR to provide assistance for that.’ (Mother of SM, 21-year-old child with cerebral palsy)

Many mothers had a positive outlook in terms of their children’s future and described how they look forward to ongoing emotional and financial support from support groups hosted by the CBR: ‘We really appreciate the emotional support from CBR staff. Although our son no longer needs therapy, we would appreciate on-going emotional support for our son’ (Mother of PP, 11-year-old child with cerebral palsy).

In addition: ‘My family and I need help with our outlook on the future. I am not sure what to expect’ (Mother of PP, 11-year-old child with cerebral palsy).

Some mothers described a bleak outlook for their child’s future describing how they are worried that their child might never become independent and that he or she will be a burden for the rest of their life.

#### Ethical considerations

Approval of this study was secured by the Duquesne University Institutional Review Board for protection of human subjects in the study. Requirements for confidentiality were also observed. Respondents who agreed to participate in this study signed a consent form to participate in the research study. The purpose, risks and benefits of the study were explained in this form as well as requirements for confidentiality. Recruitment of participants occurred as a collaborative effort between one of the co- investigators and the CBR staff. Informed consent was obtained prior to beginning the interview, organised by CBR staff. All Institutional Review Board regulations were carefully followed to protect participant confidentiality.

#### Trustworthiness and validity

The findings of this study are trustworthy as they are based on the human experience as described by all participants. This study is qualitative in nature; therefore, researchers do not guarantee that the same results would be uncovered if this study was replicated. Verification of data was also done by relating the collected data to each respondent after each interview.

## Discussion

The mothers in this study described their child’s activities as very basic and mostly taking place at home with few social activities and recreation activities taking place.

### Acceptance by family, friends and community

Mothers in this study described both how acceptance or rejection of their children by family, friends and the community impacts positively and negatively on their children’s social participation.

### Recommendations for community-based rehabilitation

These findings suggest CBR programmes might create family and community education programmes to inform and educate entire communities on the causes and implications of living with a disability. Further, such programmes involve ways of teaching community members how to support and encourage a household of a child with a disability in terms of how to be child-centred, and to help the mother engage their child in activities that are beneficial to the child’s development. If communities are made aware of the difficulties associated with living with a disability, children with disabilities and their families may have a greater chance of being accepted and thus enhance children with disabilities’ social participation and quality of life.

### Schools and future education

Although inclusive education is part of the Zambian government’s educational policy, more than half of the children in this study did not attend school. A shortage of accessible educational services in terms of children with disabilities was a major concern of all the interviewed mothers; most of them look to CBR to assist them in advocating for accessible schools and financial support for their children’s education.

### Recommendations for community-based rehabilitation

CBR programmes might consider teaching advocacy skills to families of children with disabilities to encourage families to be more proactive and to take responsibility for securing educational opportunities for their children. Further, the study observes that CBR staff members are expected to consider advocating for and with families of children with disabilities in an effort to include children with disabilities in Zambia’s education system.

### Physical burden

Mothers of children with disabilities described the physical burden of carrying their children and how this creates a barrier to taking their children to school, church or in engaging in other community activities.

### Recommendations for community-based rehabilitation

The study established that most of the children with disabilities who participate in CBR services had already received wheelchairs where appropriate. Yet, mothers expressed a desire for the CBR to purchase suitable wheelchairs for their children that can withstand the rough terrain of village roads. It might be useful for CBR to consider the following questions:

- Is it realistic to upgrade mobility equipment as a child grows older?
- Are there other nongovernmental organisations to assist in addressing equipment needs?

### Financial burden and family income

Most mothers discussed their limited family income as a barrier to their children’s social participation. Some mothers depend on family members to help pay school and medical fees. All the mothers involved in this study seek access to education programmes and general opportunities for their child that would normally be denied to their children because these opportunities cost too much money.

### Recommendations for community-based rehabilitation

Livelihood activities are part of this CBR programme. However, some mothers seek different types of livelihood activities other than those offered to them by CBR. A good example is the making of crafts or tailoring which they can do at home whilst caring for their children. CBR programmes should consider expanding livelihood programming to meet the needs of mothers who need to be at home all day because of their children’s conditions.

### Support groups and emotional support for mothers

Most mothers described CBR as a strong support for their child’s social participation. Yet, many mothers described themselves as a barrier to their child’s social participation; these mothers feel emotionally insecure and lack basic skills needed to help their child engage and participate in developmental activities.

### Recommendations for community-based rehabilitation

Findings suggest that mothers need a stronger support system. CBR might consider providing emotional and spiritual support system for mothers by expanding the support group and networks through collaborating with local churches and faith communities. Another way might be to expand the community health worker roles and responsibilities to include educating and mentoring mothers.

### Implications for future research

This research points to the need for a broad-based study to explore patterns of social participation of children with disabilities in Africa as well as the barriers and supports for social participation and effective strategies for mothers to help their children overcome these barriers. Researchers might also study contexts in which inclusive education is enforced in Africa and effective strategies to reach that goal so that all children with disabilities might have access to education that meets their needs.

### Limitations of the study

Limitations in this study include a small sample size and unequal distribution of gender amongst the interviewed mothers of children with disabilities. Another limitation is that the conclusions are not generalisable as a result of the small sample size.

Some qualitative researchers argue that validity is not applicable to qualitative research and, at the same time, realise the need for a qualifying check or measure for their research (Winter 2000). The quotations from participants validate the themes. The meanings found in the data and in the conclusions were based on evidence. The researchers used a systematic, analytic approach to uncover an accurate representation of the data collected. The rigour of this study is reflected in the use of reflexivity, particularly visible in the primary investigator who sought constant input in terms of the data analysis process and coding from the local Zambian co-researcher. The research also took into account the fact that a so-called ‘Westerner’ from a developed country conducted research within a developing country. This bias was addressed by involving the Zambian co-investigator in all aspects of the study.

## Conclusion

This study is significant in that it is one of only a few studies that explored the level of support received and/or offered and barriers experienced in terms of social participation for children with disabilities in Africa. CBR staff might consider teaching advocacy skills to all stakeholders of children with disabilities including families and communities, but especially to the children’s mothers. CBR staff might further consider collaborating with mothers and community stakeholders in an effort to develop policies that are holistic, child-centred and address the challenges experienced by children with disabilities and their families. Together, mothers can advocate for effective policies, inclusive education, and thus stronger families, communities and schools, focussing on inclusion for all.
